# Gut Microbiome Responses to Nutritional and Lifestyle Interventions in Pediatric Obesity: A Systematic Review Toward Precision Nutrition

**DOI:** 10.3390/children13060828

**Published:** 2026-06-18

**Authors:** Iuliana Margasoiu, Alin Constantin Pînzariu, Lorena Mihaela Manole, Elena-Lia Spoială, Gabriela Păduraru, Gabriela Ghiga, Irene Paula Popa, Dragomir Nicolae Șerban, Ionela Lăcrămioara Șerban, Laura Mihaela Trandafir

**Affiliations:** 1 Grigore T. Popa University of Medicine and Pharmacy Iasi, 700115 Iasi, Romania; grumezescu.iuliana@d.umfiasi.ro (I.M.); alin.pinzariu@umfiasi.ro (A.C.P.); paduraru.gabriela@umfiasi.ro (G.P.); gabriela.ghiga@umfiasi.ro (G.G.); irene-paula_popa@umfiasi.ro (I.P.P.); dragomir.serban@umfiasi.ro (D.N.Ș.); ionela.serban@umfiasi.ro (I.L.Ș.); laura.trandafir@umfiasi.ro (L.M.T.); 2 Sfânta Maria Emergency Children Hospital, 700309 Iasi, Romania

**Keywords:** gut microbiota, dietary intervention, physical activity, lifestyle intervention, prebiotic, probiotic, synbiotic, postbiotic, obesity, children

## Abstract

**Highlights:**

**What are the main findings?**

- Nutritional and lifestyle interventions in pediatric obesity were associated with measurable but heterogeneous changes in gut microbiota diversity, composition, and predicted function.
- Fiber-rich diets, prebiotic, probiotic, synbiotic, and postbiotic supplementation, calorie restriction, and physical activity were linked to taxon-specific microbial shifts, including changes in beneficial taxa such as *Bifidobacterium*, *Faecalibacterium*, *Blautia*, and *Akkermansia muciniphila*.

**What are the implications of the main findings?**

- Gut microbiome modulation may represent a promising adjunctive strategy in pediatric obesity management, but current evidence does not support its use as a stand-alone therapeutic target.
- Methodologically standardized trials are needed to determine whether microbiome changes directly contribute to clinically meaningful metabolic and anthropometric improvements.

**Abstract:**

**Background:** Childhood obesity is increasingly associated with gut microbiome dysbiosis. This systematic review (PROSPERO CRD420251131354) evaluates evidence from studies published between 2020 and 2026 assessing how nutritional and lifestyle interventions influence gut microbiota in children with obesity. **Methods:** A systematic search of PubMed, EMBASE and EBSCO identified 21 interventional studies involving children aged 5–18 years with obesity, with the last search conducted in April 2026. Interventions comprised prebiotics, probiotics, synbiotics, postbiotics, high-fiber diets, calorie-restricted dietary approaches, and lifestyle modifications such as physical activity. Microbiome outcomes were analyzed using 16S rRNA sequencing, quantitative real-time polymerase chain reaction (qPCR), or metagenomics. Risk of bias was evaluated using the RoB 2 and ROBINS-I (version 2) tools. Due to substantial heterogeneity in study design, participant characteristics, intervention types, and analytical methods, a meta-analysis was not feasible. **Results:** Across 21 studies, nutritional interventions included measurable but heterogeneous alterations in gut microbiome composition. Inulin supplementation was associated with a significant increase in alpha diversity and with higher relative abundances of *Bifidobacterium*, *Blautia*, *Megasphaera*, *Subdoligranulum*, and *Eubacterium coprostanoligenes*. Synbiotic supplementation increased *Prevotella* and *Dialister* and reduced the Firmicutes/Bacteroidetes ratio. High-fiber dietary interventions increased *Faecalibacterium*, *Bifidobacterium*, and *Clostridium*, while reducing *Bacteroides*, and were associated with shifts in metabolic pathways related to carbohydrate, lipid, and nucleotide metabolism. Calorie-restricted diets and combined diet–exercise interventions increased beneficial taxa such as *Akkermansia muciniphila*, improved microbial diversity, and correlated with favorable metabolic and anthropometric outcomes. Overall, nutritional and lifestyle interventions in pediatric obesity were associated with taxon-specific and context-dependent microbiome changes, rather than uniform restructuring. **Conclusions:** Nutritional interventions can modulate gut microbiota diversity, composition, and predicted function in pediatric obesity; however, the observed effects vary substantially across studies. The limited number of trials, small sample sizes, and methodological heterogeneity underscore the need for larger, standardized studies to better define clinical and therapeutic implications.

## 1. Introduction

Obesity is a public health issue, along with the fact that the number of overweight or obese children and teenagers has reached alarming levels [1,2].

Recent global analyses indicate that approximately 18.1% of children aged 5–14 years and 20.3% of adolescents aged 15–24 years were living with overweight or obesity in 2021, with rising trends across regions and income levels. In younger children under the age of 5, overweight prevalence is also increasing, estimated at around 5.5% in 2024 [3,4]. Teenagers and young adults will develop early conditions including type 2 diabetes and metabolic dysfunction-associated steatotic liver disease (MASLD), or high blood pressure [5]. In addition, self-esteem and quality of life are negatively impacted [6,7]. Since these pathologies are progressive, their onset is only a matter of time.

Statistics have shown that almost 90% of people who were obese during childhood or adolescence were likely to continue to have weight problems into adulthood. The fast increase in childhood obesity prevalence has emerged as a key factor in enhancing obesity cases among adults [8,9]. Therefore, the effective fight against childhood obesity is of major importance in the current context.

The development of obesity in children is influenced by a variety of factors, including genetics, endocrine factors and eating behaviors. However, for the most part, excessive energy intake and sedentary behavior are the main determinants of excess weight [10]. This is why current treatment strategies in the pediatric population include dietary interventions and physical activity as lifestyle changes [11,12].

Obesity has been associated with changes or alterations in the gut microbiota [13,14], and scientific data is increasingly emerging in this regard. Studies conducted on human subjects have highlighted significant differences in the composition and diversity of the gut microbiota between obese and non-obese individuals [15,16,17]. Among the most important observations, there was a lower abundance of bacteria from the Bacteroidetes and Actinobacteria species, and a higher abundance of the Firmicutes species in obese individuals [15,18].

Emerging studies highlight that gut microbiota has been increasingly recognized as a key contributor to the development of childhood obesity in children [19]. Results related to dysbiosis have shown increases in the abundance of the species *Akkermansia muciniphila*, *Clostridium difficile*, *Bacteroides plebeius*, *Bacteroides eggerthii*, *Enterococci*, *Blautia*, *Sutterella*, and *Klebsiella*. Furthermore, the species *Akkermansia muciniphila* and *Desulfovibrio* were significantly associated with BMI [20,21,22,23].

Nevertheless, despite the increasing interest, there are still significant uncertainties with regard to the impact of nutritional intervention on the gut microbiome in pediatric patients with excess weight. In addition to nutritional interventions based on specific diets, prebiotics, probiotics, synbiotics, and postbiotics, other lifestyle factors, such as physical activity, may modify the microbial balance in children with obesity or who are overweight. The current literature in this field shows substantial variability in terms of study design, characteristics of the population analyzed, type of intervention or the methods used in microbiome analysis, resulting in inconsistent and sometimes conflicting findings. Owing to these methodological differences, it is difficult to establish whether specific dietary or lifestyle strategies influence gut microbiome composition or function, or whether the observed microbial shifts translate into clinically meaningful changes. Thus, the aim of this systematic review is to critically assess recent evidence on how nutritional and lifestyle interventions influence gut microbiome composition and function in pediatric patients with obesity, and to pinpoint current limitations, inconsistencies or significant gaps in the literature that may justify further investigation.

## 2. Materials and Methods

### 2.1. Protocol Registration and Reporting

This systematic review was conducted in accordance with the PRISMA guidelines (Preferred Reporting Items for Systematic Review and Meta-Analysis) adapted from Page MJ et al. [24]. A structured search protocol was elaborated to evaluate evidence from studies published between 2020 and 2026 investigating how nutritional interventions influence the gut microbiota in children with obesity. The protocol was registered in PROSPERO (CRD420251131354).

### 2.2. Search and Selection Process

**Figure 1:**
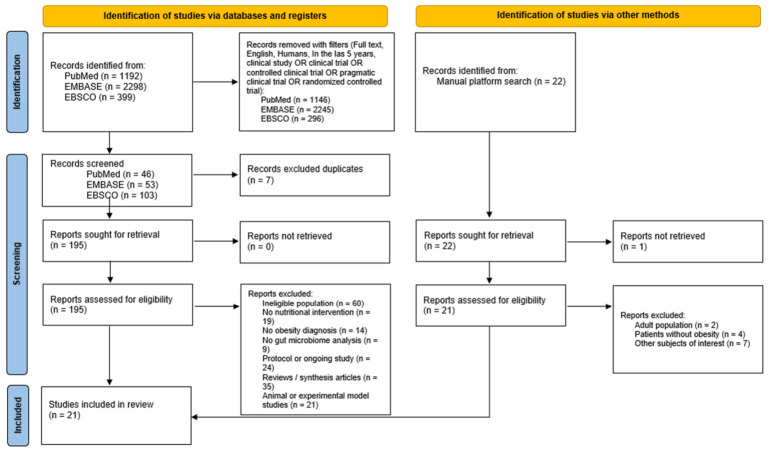
PRISMA flowchart, adapted from Page MJ et al. [24].

The PubMed, EMBASE, and EBSCO databases were used in the selection of the relevant literature by inputting both combined controlled vocabulary (MeSH for PubMed, Emtree for EMBASE) and free-text terms. The search strategy relied on the following keywords: (‘obesity’ OR ‘pediatric obesity’ OR ‘childhood obesity’) AND (‘pediatric’ OR ‘child’ OR ‘adolescent’ OR ‘children’ OR ‘adolescents’) AND (‘gut microbiota’ OR ‘gut microbiome’ OR ‘gut flora’ OR ‘intestinal flora’ OR ‘intestinal microbiome’) AND ((‘nutrition’ OR ‘nutritional’ OR ‘diet’ OR ‘diet therapy’ OR ‘nutrition therapy’) OR (‘physical activity’ OR ‘exercise’)). Search strategies were adapted to the specific requirements of each database. We included only English-language studies conducted in human pediatric populations. Study selection and reporting followed the PRISMA guidelines, using the specific checklist and flow diagram to support transparency and reduce the risk of bias (Figure 1).

The aim was to pinpoint studies on pediatric obesity that evaluate nutritional intervention and its impact on the gut microbiome. We applied filters to include full-text articles published in English, involving human subjects, from the last five years (January 2020 to April 2026), and limited the search to clinical studies, including clinical trials, controlled clinical trials, and randomized and non-randomized trials.

The PRISMA checklist and flow diagram were employed to mitigate the risk of error and to secure a high-quality comprehensive search strategy. In the case of studies that returned duplicate datasets across the databases used, a single study was included.

All decisions concerning study inclusion or exclusion were made by two authors, and any discrepancies were resolved through consensus in accordance with predefined inclusion and exclusion criteria.

The inclusion criteria were the following: randomized or non-randomized controlled trials, interventional studies (with or without a control group), prospective cohort studies with intervention, studies including the pediatric population (children and adolescents aged 5–18 years) diagnosed with obesity, studies analyzing the effect of nutritional and lifestyle interventions on the gut microbiome, studies written in English with full text available, and studies published between 2020 and April 2026.

Exclusion criteria included: review articles or case reports, studies conducted on laboratory animals or adults, studies including children without an obesity diagnosis, studies that did not analyze the gut microbiome, studies that did not investigate the effect of a nutritional intervention, and studies presenting topics irrelevant to the research objective.

### 2.3. Data Extraction

The titles, abstracts, and full texts of relevant studies were systematically reviewed by two independent researchers. Eligible articles were assessed independently to verify methodological quality and data accuracy.

The extracted data was entered into a database using Microsoft Excel software, where the following variables were included: author’s name, year of publication, country, study design, population, type and duration of nutritional intervention, main results, microbiome analysis method, and additional comments.

### 2.4. Risk-of-Bias Assessment

The methodological quality and risk of bias of the included studies were independently evaluated by two reviewers using validated assessment tools. Randomized studies were assessed using the RoB version 2 tool (Revised Cochrane Risk-of-Bias Tool for Randomized Trials), while non-randomized interventional studies were evaluated using the ROBINS-I V2 tool (the Risk of Bias in Non-randomized Studies–of Interventions, Version 2 assessment tool). The RoB 2 tool evaluates potential bias across domains related to the randomization process, deviations from intended interventions, missing outcome data, outcome measurement, and selective reporting, providing an overall risk-of-bias judgment. The ROBINS-I version 2 tool assesses bias related to confounding, intervention classification, participant selection, deviations from intended interventions, missing data, outcome measurement, and selective reporting.

Each domain was rated according to predefined methodological criteria. Any disagreements between reviewers were resolved through discussion and consensus, and consultation with a third reviewer was not required.

## 3. Results

Following the systematic literature search conducted for studies published between 2020 and 2026, a total of 257 records were initially identified. After scanning the titles and abstracts, removing duplicates, and excluding studies that did not meet the relevance criteria, 21 articles ultimately met the inclusion criteria.

### 3.1. Prebiotic Interventions

After the literature review, six randomized controlled trials evaluating the effects of prebiotic supplementation in pediatric patients with obesity were identified (Table 1). These articles reported distinct primary outcomes related to anthropometric parameters, metabolic profiles, and gut microbiota composition in children and adolescents who were overweight or obese.

Six-month inulin supplementation in children aged 7–15 years was associated with significant modulation of gut microbiota composition, including increased alpha diversity and enrichment of beneficial taxa such as *Bifidobacterium*, *Blautia*, and *Megasphaera*, as well as other butyrate-producing bacteria (*Agathobacter*, *Eubacterium coprostanoligenes*, and *Subdoligranulum*) [29]. Using Phylogenetic Investigation of Communities by Reconstruction of Unobserved States 2 (PICRUSt) for inference of microbial functional pathways, the study by Visuthranukul et al. identified differences in predicted microbial pathways, including those related to riboflavin metabolism and proteasome function. However, these functional predictions were correlational and were not validated through direct metabolomic or transcriptomic measurements. In addition, a secondary metabolomic analysis of a randomized cohort demonstrated that inulin intake also influenced gut microbiota-derived metabolites involved in the gut–brain axis, with significant increases in putrescine, spermine, and tyrosine, and correlations between microbial shifts and changes in these bioactive compounds [25]. Aksornkitti et al. reported substantial temporal variability in gut microbiome composition among children with obesity over a 6-month intervention period, characterized by frequent intra-individual enterotype switching. Although overall shifts in enterotype distribution were observed over time, no consistent intervention-specific effects attributable solely to inulin supplementation were identified [27].

In a separate randomized trial conducted by Panichsillaphakit et al., inulin supplementation was associated with changes in eating behavior parameters, particularly a reduction in emotional undereating [26]. Gut microbiome analyses were exploratory, with no statistically significant changes in alpha diversity or overall community-level structure reported as primary outcomes. The authors pointed out correlations between selected microbial taxa (*Agathobacter* and members of *Lachnospiraceae* spp.) and eating behavior measures. However, these associations were not interpreted as direct, intervention-driven effects on the gut microbiota, and no causal relationships were established.

Prebiotic supplementation with chitosan administered for 12 weeks in adolescents aged 10–18 years resulted in a significant reduction in BMI z-score and favorable alterations in gut microbiota composition, characterized by a decrease in Firmicutes and the Firmicutes/Bacteroides ratio, alongside an increase in *Bacteroides* and *Akkermansia*, compared with placebo [28].

### 3.2. Synbiotic Interventions

Synbiotic supplementation (Table 2) was evaluated in a randomized controlled trial by Kilic Yildirim et al. [30]. The intervention induced taxon-specific changes at both genus and species levels, including increased abundance of *Prevotella*, *Dialister*, *Coprococcus*, and selected Lachnospiraceae. However, no significant increase in alpha diversity or distinct community-level clustering was observed, and microbial shifts were primarily identified through group-level comparisons [30]. Overall, these findings indicate a selective taxonomic modulation rather than a comprehensive restructuring of the gut microbiome. In another randomized, double-blind interventional trial, Martínez-Martínez et al. evaluated probiotic yogurt alone compared with synbiotic formulations containing either inulin or Agave salmiana fructans in children who were overweight or obese, reporting gut microbial modulation. There were modifications in beta diversity and genus-level compositional shifts, including decreased *Blautia* and *Holdemanella* and increased *Faecalibacterium* and *Intestinibacter*, while anthropometric improvements were modest and primarily observed in the synbiotic groups [31].

### 3.3. Probiotic Interventions

Three studies evaluated probiotic-based interventions targeting the gut microbiome in pediatric populations who were overweight or obese (Table 3). In a randomized, double-blind, placebo-controlled trial, Chen et al. reported that 12-week supplementation with a multi-strain probiotic in children who were overweight or obese was associated with greater reductions in body weight and BMI. These changes were accompanied by favorable improvements in lipid profile and modulation of gut microbiota composition, compared with placebo [32]. In another cross-over trial, Solito et al. demonstrated that eight-week supplementation with *Bifidobacterium breve* improved insulin sensitivity indices in children and adolescents with obesity and insulin resistance. Modest reductions in waist circumference and fasting insulin were also observed, although there were no significant changes in body weight. Microbiome assessment showed limited compositional alterations in bacterial composition, while functional changes in SCFA profiles were observed [33]. In a randomized, double-blind, placebo-controlled pilot trial, Verma et al. evaluated multi-strain probiotic supplementation (Visbiome^®^, ExeGi Pharma LLC., Gaithersburg, MD, USA) in adolescents with severe obesity and reported no significant changes in overall gut microbial diversity or taxonomic composition after 12 weeks of treatment, although a non-significant reduction in the Firmicutes/*Bacteroides* ratio and a significant improvement in fasting glucose levels were observed in the probiotic group compared with placebo [34].

### 3.4. Postbiotic Interventions

One randomized clinical trial investigated a microbiome-targeted postbiotic intervention in pediatric obesity. Coppola et al. conducted a quadruple-blind, placebo-controlled trial in children and adolescents aged 5–17 years with obesity, in which oral sodium butyrate supplementation (20 mg/kg/day) was administered as an adjunct to standard lifestyle care for six months. Compared with placebo, butyrate supplementation resulted in a significantly higher proportion of participants achieving a clinically meaningful reduction in BMI standard deviation (SD) score (≥0.25 SD) [35]. Per-protocol analyses demonstrated greater reductions in BMI, BMI z-score, and waist circumference, as well as significant improvements in fasting insulin, HOMA score, ghrelin, interleukin-6, and micro-RNA-221 expression in the butyrate group. Gut microbiome analysis did not reveal significant between-group differences in overall taxonomic composition. However, baseline microbiome signatures were associated with metabolic response to the postbiotic intervention. The main results of postbiotic interventions in pediatric obesity are summarized in Table 4.

**Table 3:** The main results of probiotic intervention in pediatric obesity.

Author; Year; Country	Study Design	Population (Age, Diagnosis)	Type of Intervention	Duration of the Intervention	Main Results	Microbiome Analysis Method	Comments
Chen et al., 2022 [32]; China	RCT (double blind, placebo-controlled clinical trial)	*n* = 56, Children 6–12 years with overweight and obesity	Probiotic supplementation (*Lactobacillus salivarius* AP-32, *L. rhamnosus* bv-77, and *Bifidobacterium animalis* CP-9), plus standardized diet and exercise guidance vs. placebo	12 weeks	↑ *Bifidobacterium*, ↑ *Lactobacillus*, partial correction of obesity-associated dysbiosis; probiotic group showed greater reductions in body weight and BMI, with improvements in lipid profile (↓ total cholesterol, ↓ LDL, ↑ HDL) and increased adiponectin levels	16S rRNA gene sequencing (Illumina); functional prediction using PICRUSt	Lifestyle intervention applied to both groups limits attribution of effects solely to probiotics
Solito et al., 2021 [33]; Italy	RCT (double-blind, placebo-controlled cross-over trial)	*n* = 101 Children and adolescents 6–18 years with obesity and insulin resistance	Probiotic supplementation with *Bifidobacterium breve* BR03 and B632 (2 × 10^9^ colony-forming units (CFU)/day) + standardized Mediterranean diet and physical activity	8 weeks (first phase analyzed due to carry-over effect)	Probiotics improved insulin sensitivity compared to placebo; modest additional effects on waist circumference and fasting insulin; no major changes in inflammatory markers	qPCR quantification of selected bacterial groups; fecal SCFA profiling by gas chromatography and mass spectrometry	Metabolic benefits exceed anthropometric effects; microbiota functionality rather than composition may present modifications; cross-over design limited by carry-over effect
Verma et al., 2021 [34]; USA	RCT (double blind, placebo-controlled pilot trial)	*n* = 15 (probiotic *n* = 8 vs. placebo *n* = 7), adolescents 13–19 years old with severe obesity	Multi-strain probiotic supplementation (Visbiome^®^, containing *Lactobacillus*, *Bifidobacterium*, and *Pediococcus strains*; 2 sachets/day) vs. isocaloric placebo; no concurrent dietary or physical activity intervention allowed	12 weeks	No significant changes were observed in alpha diversity or beta diversity at either genus or phylum level. The Firmicutes/*Bacteroides* ratio showed a quantitative decrease in the prebiotic group; no significant between-group differences were observed for insulin, HOMA-IR, body weight, BMI, BMI z-score or fecal calprotectin	Shotgun metagenomic sequencing (Illumina NextSeq 500)	Pilot study with very small sample size and high participant loss rate; limited statistical power for microbiome outcomes; effects on gut microbiota were modest and not statistically significant; clinical metabolic outcomes were secondary endpoints

BMI = Body mass index, CFU = Colony-forming units, PICRUSt = Phylogenetic Investigation of Communities by Reconstruction of Unobserved States, RCT = Randomized controlled trial, rRNA = ribosomal RNA, and qPCR = Quantitative real-time polymerase chain reaction, ↑ indicates an increase and ↓ indicates a decrease in the abundance of the respective bacterial taxa.

**Table 4:** The main results of postbiotic intervention in pediatric obesity.

Author; Year; Country	Study Design	Population (Age, Diagnosis)	Type of Intervention	Duration of the Intervention	Main Results	Microbiome Analysis Method	Comments
Coppola et al., 2022 [35]; Italy	RCT (quadruple-blind, placebo-controlled clinical trial)	*n* = 54 Children 5–17 years with obesity	Oral sodium butyrate (20 mg/kg/day) as adjunct to standard lifestyle care (Mediterranean diet and physical activity)	6 months	A higher rate of clinically meaningful improvement in BMI (≥0.25 SD) in butyrate group vs. placebo (96% vs. 56%); improvements in waist circumference, insulin resistance, ghrelin, IL-6; microbiome baseline signatures predicted metabolic response	Shotgun metagenomic sequencing; taxonomic and functional profiling (MetaPhIan); microbiome–metabolic analysis	Strong evidence for microbiome-targeted nutritional intervention; effects likely mediated by microbial metabolites; limited sample size and adherence issues noted

BMI = Body mass index, RCT = Randomized controlled trial.

### 3.5. Dietary and Combined Lifestyle Interventions

Eight prospective, non-pharmacological intervention studies evaluated the effects of dietary or combined lifestyle interventions on anthropometric outcomes and gut microbiota composition in children and adolescents with obesity (Table 5). All studies utilized pre–post designs without randomization, with intervention durations ranging from six weeks to three months.

Across these studies, dietary modification alone or in combination with structured physical activity was consistently associated with improvements in anthropometric parameters, particularly reductions in body weight, BMI, waist circumference, or body fat percentage. Specifically, individualized lifestyle modification and calorie-restricted, fiber-enriched dietary interventions resulted in significant shifts in gut microbiota composition, including increased relative abundance of taxa such as *Bifidobacterium*, *Blautia*, *Akkermansia muciniphila*, and other SFCA-producing genera, alongside reductions in obesity-associated taxa such as *Bacteroides* and *Megamonas* [36,37,38,39]. Changes in microbial alpha diversity were heterogeneous, with some studies reporting significant increases following intensive diet and exercise programs [38,39], while others observed no significant diversity changes after shorter or less intensive interventions [40]. In the smallest pilot study, overall microbial diversity remained unchanged, but specific genus-level changes, particularly increased *Odoribacter* abundance, were significantly associated with reductions in waist circumference [40]. In a non-randomized lifestyle intervention trial, Lee et al. reported that participants classified as responders exhibited significant reductions in BMI, body fat percentage, and alanine aminotransferase levels, accompanied by metabolomic changes involving branched-chain amino acid, purine, and fatty acid biosynthesis pathways, with additional correlations between microbial taxa and metabolic markers [41]. In a randomized controlled dietary intervention targeting free sugar restriction, Cohen et al. observed significant reductions in hepatic fat alongside metabolomic pathway modulation related to amino acid and lipid metabolism, while microbiome alterations were limited and did not remain statistically significant after multiple testing correction [42]. In a pilot randomized controlled study, Aqeel et al. demonstrated that a dietitian-guided food-provisioning intervention significantly improved diet quality by increasing fiber and whole-grain intake and reducing dairy consumption, although no significant changes were observed in anthropometric outcomes or gut microbiome composition [43].

### 3.6. Physical Activity

According to Quiroga et al., a 12-week combined strength and endurance exercise program partially reversed obesity-related microbial alterations in children. Specifically, the comparison of fecal microbiota profiles between healthy control children and obese participants aged between 7 and 12 years old after the exercise intervention revealed that the trained obese group exhibited microbial community clustering similar to the healthy controls. Physical activity was associated with a significant reduction in Proteobacteria and Gammaproteobacteria, taxa commonly linked to metabolic dysfunction, alongside increases in beneficial genera such as *Blautia*, *Dialister*, and *Roseburia* [44]. These results are reinforced by Morgado et al., who found that a 12-week physical activity intervention in overweight and obese children led to distinct gut microbiota changes, reinforcing the notion that approximately three months of exercise may be sufficient to induce microbiome modulation in pediatric obesity [45]. Taken together, these findings suggest that physical activity can beneficially reshape the gut microbiota in obese children and may contribute to reduced obesity-associated inflammation through microbiota-mediated mechanisms [46].

The following Table provides an overview of studies examining how different physical activity interventions influence gut microbiota composition in pediatric populations, illustrating the shifts toward a healthier microbial profile described above (Table 6).

**Table 5:** The main results of dietary and combined lifestyle interventions in pediatric obesity.

Author; Year; Country	Study Design	Population (Age, Diagnosis)	Type of Intervention	Duration of the Intervention	Main Results	Microbiome Analysis Method	Comments
Cho KY et al., 2021 [36]; South Korea	Prospective longitudinal lifestyle pre–post intervention study	*n* = 36, Children and adolescents 7–18 years with obesity	Individualized lifestyle modification program including nutritional counseling + physical activity	8 weeks	19 individuals gained fat mass; 17 individuals lost fat mass; In children who lost weight: ↑ *Firmicutes*, ↓ Bacteroidetes. There were changes also in microbiome function. Children with fat loss showed significant changes in gut microbiota composition, reduced richness, and altered predicted metabolic pathways. Children who gained weight showed opposite changes.	16S rRNA gene sequencing (Illumina MiSeq)	Non-randomized design without control group; heterogeneous intervention; provides mechanistic insight into microbiota dynamics associated with lifestyle-induced weight change
Zhou M et al., 2024 [37]; China	Prospective before–after dietary intervention study with control group	*n* = 30 children with obesity vs. *n* = 40 healthy children; Children school-aged 6–12 years	Personalized calorie-restricted diet (balanced energy-restricted diet with increased dietary fiber, moderate protein, and controlled fat intake)	12 weeks	Significant decrease in weight, BMI, BMI-z score, body fat percentage; favorable microbiome changes: ↑ beneficial bacteria (e.g., *Bifidobacterium*, *Lactobacillus*), ↓ *Bacteroides* and ↑ *Megamonas* microbial diversity; positive correlation between microbiome changes and metabolic parameters	16S rRNA gene sequencing (Illumina NovaSeq)	Single-arm pre–post design without randomized obese control group; dietary intake self-reported; lifestyle counseling provided concurrently; microbiome changes are associative
Peng LJ et al., 2024 [38]; China	Prospective before–after combined dietary and exercise intervention (DEI)	*n* = 86 children with obesity, of whom 39 underwent intervention vs. *n* = 107 healthy controls; children 6–12 years	Diet + exercise for weight loss: calorie-restricted, high-fiber balanced diet (1300–1500 kcal/day) plus supervised aerobic exercise (~4 h/day, 6 days/week)	6 weeks	DEI group resulted in significant weight loss and improvement in dysbiosis. Alpha diversity indices increased significantly after intervention. Gut microbiota composition shifted at the individual level, with ↑ abundance of *Akkermansia muciniphila*, Rikenellaceae, and Enterobacteriaceae, and ↓ *Sutterella* and *Alcaligenaceae*	16S rRNA sequencing (Illumina MiSeq)	Non-randomized, single-arm intervention; short intervention duration; intensive camp-based lifestyle program limits generalizability; microbiome changes are associative rather than causal
Morán-Ramos et al., 2022 [40]; Mexico	Pilot intervention study, no control	*n* = 6; Children 11–14 years with obesity	Multidimensional lifestyle intervention: hypocaloric individualized diet + nutritional recommendations + physical activity	6 weeks	↓ Waist circumference after the intervention, while BMI, BMI z-score, body fat percentage, and metabolic parameters remained unchanged; overall gut microbiota composition and alpha diversity did not change; the reduction in waist circumference was significantly associated with an ↑ *Odoribacter* abundance	16S rRNA gene sequencing (Illumina MiSeq)	Very small sample size, single-arm design without obese control group, short intervention duration; findings are exploratory and associative, limited generalizability
Huang et al., 2020 [39]; China	Prospective before–after lifestyle intervention study	*n* = 24; adolescents 9–16 years with obesity	Combined lifestyle intervention: Hypocaloric diet (~60% carbohydrates, 20% protein, 20% fat) + intensive endurance and strength exercise program (~5 h/day, 6 days/week)	6 weeks	Significant decrease in weight, BMI, body fat mass, waist-to-hip ratio; significant improvements in central hemodynamic parameters (↓ AIx75, ↓ resting heart rate); gut microbiota analysis showed decreased Firmicutes/Bacteroidetes ratio, increased alpha diversity, ↓ abundances of Lactobacillales, Streptococcaceae, *Veillonella*, and ↑ abundances of Lentisphaeria, Christensenellaceae, Butyricimonas, *Victivallis*; microbiota changes were significantly correlated with hemodynamic, anthropometric, and metabolic parameters	16S rRNA sequencing (Illumina MiSeq)	Non-randomized, single-arm design without obese control group; highly intensive camp-based intervention limits generalizability; short intervention duration; microbiome findings are associative and hypothesis-generating
Lee et al., 2023 [41]; South Korea	Non-randomized clinical trial with before–after lifestyle intervention and responder analysis	*n* = 17 responders; *n* = 19 non-respondrs; *n* = 22 normal-weight children included as reference group	Individualized multidisciplinary lifestyle intervention including dietary modification, physical activity counseling, and behavioral monitoring supervised by pediatric clinicians, dietitians, and exercise specialists	8 weeks	↓ *Bacteroides* and changes in Firmicutes/*Bacteroides* balance in post-intervention responders; significant reductions in BMI, total body fat percentage, whereas non-responders showed increases in body weight and BMI; baseline metabolomic analysis showed elevated branched-chain amino acids, purine metabolism markers, and altered bile acid profiles in obese participants	16S rRNA gene sequencing (Illumina MiSeq)	Multi-omics exploratory study with small sample size and short intervention duration; absence of randomization limits causal inference; microbiome results partially derived from previously published dataset using the same cohort; strong mechanistic insight but limited external generalizability
Cohen et al., 2023 [42]; USA	RCT (secondary multi-omics analysis)	*n* = 40, adolescent boys aged 11–16 years with biopsy-proven NAFLD, with overweight or obesity	Provision of a low free-sugar diet (<3% of total energy intake from free sugars) compared with habitual diet	8 weeks	Microbiome analysis showed increased richness at the phylum level and taxon-specific ↑ in *Ruminococcus bromii* and *Phascolarctobacterium*, although no microbiome findings remained significant after multiple testing correction; the low free-sugar diet resulted in a significant reduction in hepatic fat compared with usual diet	16S rRNA sequencing (Illumina MiSeq)	Microbiome results derived from small sub-sample; study designed primarily to evaluate hepatic fat reduction rather than microbiome modulation; homogeneous male-only cohort limits generalizability; exploratory multi-omics findings require validation
Aqeel et al., 2025 [43]; USA	RCT (pilot trial)	*n* = 33 (intervention *n* = 17), children aged 6–11 years with obesity	Intensive health behavior and lifestyle treatment in both groups; intervention arm additionally received dietitian-guided grocery food provisioning, promoting a high-fiber, plant-focused, low-dairy dietary pattern	4-week intervention with follow-up at 8 weeks	No significant changes were observed in body weight, food insecurity status, or gut microbiome diversity; some beneficial microbiome taxa trends were described but did not reach statistical significance; the intervention significantly improved diet quality compared with usual care, with increased intake of whole grains and fiber and reduced dairy consumption	16S rRNA (Illumina MiniSeq)	Study primarily evaluated feasibility and dietary behavior modification rather than weight reduction or microbiome modulation

AIx75 = Augmentation index standardized to a heart rate of 75/min, BMI = Body mass index, DEI = Combined dietary and exercise intervention, rRNA = ribosomal RNA, ↑ indicates an increase and ↓ indicates a decrease in the abundance of the respective bacterial taxa.

**Table 6:** Key gut microbiome outcomes after physical activity intervention.

Author; Year; Country	Study Design	Population (Age, Diagnosis)	Type of Intervention	Duration of the Intervention	Main Results	Microbiome Analysis Method	Comments
Quiroga et al., 2020 [44], Spain	RCT (exercise intervention)	*n* = 39 children 7–12 years old with obesity vs. *n* = 14 healthy controls	Strength and endurance combined training program (2–3 sessions/week; ~45 min/session); no caloric restriction, standardized healthy lifestyle advice	12 weeks	↓ Proteobacteria phylum and Gammaproteobacteria class ↑ *Blautia*, *Dialister*, and *Roseburia*, shifting the microbiota toward a pattern observed in healthy children; exercise significantly downregulated obesity-associated inflammatory signaling pathways, including reduced NLRP3 inflammasome, CASP-1, and osteopontin expression	16S rRNA sequencing (Illumina MiSeq)	No dietary intervention or caloric restriction; modest anthropometric effects; demonstrates microbiome and inflammatory benefits of exercise independent of adiposity change
Morgado et al., 2023 [45]; Portugal	non-RCT	*n* = 15, Children 7–10 years with overweight or obesity	Recreational football (2 sessions/week; 60 min/session) alone (Football Group) or combined with structured nutrition (Nutrition and Football Group)	12 weeks	↓ *Bifidobacterium* genera across all participants; ↓ *Roseburia* in the Football Group; no significant changes in alpha diversity and body weight; significant within-group reductions were reported for BMI, BMI z-score, and waist-to-height ratio in both groups	16S rRNA sequencing (Illumina MiSeq)	Small sample size; absence of randomization; no normal-weight control group; microbiome changes were limited

RCT = randomized controlled trial; CASP-1 = antibodies against caspase-1, NLRP3 = NLR family pyrin domain containing 3, rRNA = ribosomal RNA, ↑ indicates an increase and ↓ indicates a decrease in the abundance of the respective bacterial taxa.

An important aspect of these findings is that the effects of physical activity on the gut microbiota do not appear to be solely attributable to caloric restriction or weight reduction. In the study by Quiroga et al., exercise-induced microbial shifts occurred in the absence of dietary restriction, suggesting that physical activity itself may directly influence gut microbial ecology [44]. Proposed mechanisms include exercise-mediated alterations in intestinal transit time, gut perfusion, bile acid metabolism, immune regulation, and the production of host-derived metabolites that shape the intestinal environment [44]. These pathways may promote the expansion of taxa associated with anti-inflammatory and metabolically favorable functions independently of changes in energy intake or adiposity. Therefore, exercise should be considered not only as a strategy for increasing energy expenditure but also as a potential modulator of the gut microbiota through distinct biological mechanisms.

In summary, exercise regimens in obese children generally shifted gut microbiota toward a healthier profile: reductions in obesity-associated *Proteobacteria* and increases in beneficial Firmicutes and Verrucomicrobia species were observed [44,45]. However, results were not entirely uniform, as one trial reported decreases in some commensals (e.g., *Bifidobacterium*, *Roseburia*) following exercise [45].

### 3.7. Risk-of-Bias Assessment Results

**Figure 2:**
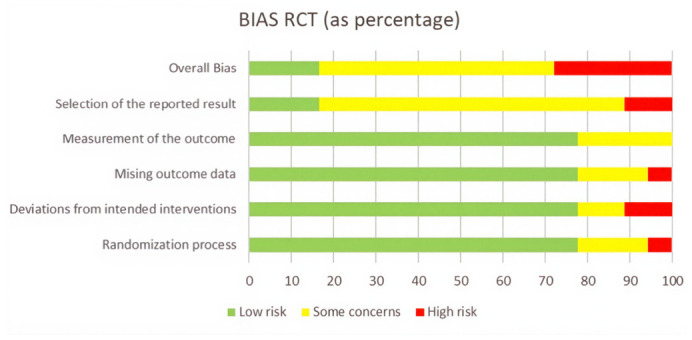
Overall risk of bias for RCT studies using RoB 2.

**Figure 3:**
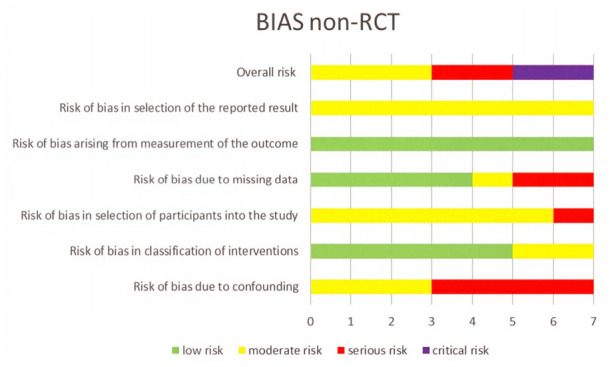
Overall risk of bias for non-randomized studies using ROBINS-I V2.

Two authors independently assessed the risk of bias using the RoB 2 tool (Revised Cochrane Risk-of-Bias Tool for Randomized Trials) [47] and the ROBINS-I V2 tool (the Risk of Bias in Non-randomized Studies–of Interventions, Version 2 assessment tool) [48]. The RoB 2 tool evaluates five key areas: bias related to the randomization process; bias resulting from departures from the intended interventions (reflecting the effect of assignment to the intervention); bias due to incomplete outcome data; bias in the measurement of outcomes; bias arising from selective reporting of results; and the overall risk of bias. In contrast, ROBINS-I V2 assesses seven domains, which include bias due to confounding, bias in the classification of interventions, bias in the selection of participants for the study or analysis, bias from deviations from intended interventions, bias caused by missing data, bias related to outcome measurement, and bias in the selection of reported results. Each domain is rated according to a predefined set of criteria that take methodological aspects into account. Consensus was reached between the two reviewers for all assessments, and it was not necessary to involve a third reviewer to resolve discrepancies. The results of overall risk of bias are summarized in Figure 2 and Figure 3.

The randomized controlled trials showed an overall moderate methodological quality. Of the controlled trials included, one was judged to have a low overall risk of bias [35] and four were considered at high risk of bias [25,31,34,43], mainly because of the deviations from intended interventions [34,43], missing outcome data [34], and selection of the reported result [31,43]. The remaining ones were classified as presenting some concerns [26,27,28,29,30,32,33,42,44] across one or two of the variables.

The non-randomized studies generally had a higher risk of bias. The risk of bias assessment using ROBINS-I V2 revealed methodological heterogeneity across included studies. Overall, only three studies were judged to have a moderate risk of bias [36,38,39] while the remaining four were judged as having some concerns regarding the risk of bias due to confounding in selection of participants or due to missing data, placing them at serious risk [37,45] and critical risk [40,41]. Outcome measurement was consistently assessed as low-risk across all studies, and classification of intervention generally posed limited concerns.

Given the differences in study design, the participants’ characteristics, and outcome measurement instruments, a meta-analysis was not feasible. Data in each study were stratified by subgroup and assessment approach to assess the precision and validity of the measurements. All extracted data were manually compiled to provide a detailed and structured summary of the results, organized by study populations and methodological features.

## 4. Discussion

This study underlines the heterogeneity and context-dependent nature of gut microbiome responses to nutritional and lifestyle interventions in pediatric patients with obesity. While multiple studies report associations between intervention-induced changes in diet or behavior and microbiota composition, the strength, consistency, and mechanistic relevance of these findings remain limited. Our investigation suggests that nutritional and lifestyle interventions can modulate the gut microbiome in children with obesity, but the direction and magnitude of changes are highly variable across studies. Baseline differences between obese and normal-weight children are also inconsistent: some studies suggest lower alpha diversity and altered community structure in overweight children [49,50,51,52], while others reported no significant differences [15,53,54,55]. Beta diversity (between-sample community differences) was likewise inconsistent; some interventions shifted community composition significantly [17], while others did not [53].

Prebiotic and synbiotic interventions demonstrated selective modulation of specific microbial taxa, rather than uniform improvements in global microbiome diversity. Notably, only one well-controlled RCT reported a statistically significant increase in alpha diversity [29], whereas other trials primarily described taxon-level or correlational findings. Functional pathway alterations were inferred from predictive models and should not be interpreted as direct evidence of metabolic reprogramming. When multiple publications appeared to originate from the same or partially overlapping study population, they were treated as related reports rather than as independent intervention cohorts. This was particularly relevant for the Thai inulin supplementation publications [25,26,27,29], which shared similar eligibility criteria, intervention arms, study duration, setting, and overlapping author groups. These reports were synthesized according to their specific outcomes, but they were not used to cumulatively inflate the number of independent participants or independent intervention trials. Therefore, conclusions derived from these publications were interpreted cautiously and were presented as complementary findings from related analyses rather than as separate replications across independent cohorts.

Dietary interventions showed substantial variability in microbiome outcomes, even when anthropometric improvements were consistent. High-fiber diets induced strain-level adaptations in specific taxa; however, these effects were population-specific and cannot be generalized across pediatric obesity phenotypes. Importantly, improvements in BMI or metabolic markers frequently occurred independently of consistent microbiome diversity changes, suggesting that microbiota modulation may not be essential for short-term clinical benefit.

Interventions combining diet and physical activity further highlighted the heterogeneity of the microbiome. Differing responses based on fat-loss/fat-gain trajectories highlighted the strong influence of host metabolic adaptation and baseline phenotype on the microbiome dynamics. A number of studies relied on correlational analyses or lacked adequate control groups, which limited causal inference.

A critical limitation across the reviewed literature is the reliance on 16S rRNA sequencing and inferred functional analyses, with limited use of shotgun metagenomics, metabolomics, or longitudinal causal modeling. Study comparability is further restricted by small sample sizes, short follow-up periods, and inconsistent outcome measures.

It is important to note that evidence from adult or non-pediatric populations cannot be directly extrapolated to children, considering the plasticity and resilience of pediatric gut microbiome development. Studies such as that carried out by Fragiadakis et al. highlight the resilience of the microbiome and transient responses rather than sustained alterability, underlining the need for caution in interpreting the effects of interventions [56].

Common taxa influenced by interventions included increases in SCFA-producing genera and health-associated microbes, along with decreases in opportunistic or pro-inflammatory taxa. Across trials, *Bifidobacterium* and other beneficial genera often rose after fiber- or biotic-based interventions [57], in accordance with their known prebiotic effects. Increased SCFA production was frequently involved: prebiotic fibers selectively fueled *Bifidobacterium* and *Lactobacillus*, elevating acetate, propionate and butyrate levels that promote satiety hormone release and improve metabolism [58]. Conversely, taxa linked to dysbiosis tended to decline. For instance, Luo et al. noted that elevated Enterobacteriaceae, Prevotellaceae and Veillonellaceae have been implicated in obesity by driving low-grade inflammation and dyslipidemia [19].

Notably, the microbiome changes did not uniformly translate to clinical improvements. For example, Nicolucci et al. [59] observed that prebiotic-induced blooms of *Bifidobacterium* and lactobacilli were associated with improved insulin sensitivity and reduced fat mass. However, a systematic analysis noted mixed correlations: in some cases, the relative abundance of Firmicutes or *Bacteroides fragilis* positively correlated with BMI, while Bacteroidetes and *Akkermansia muciniphila* were negatively correlated with BMI z-scores [46]. These divergent findings underscore that associations between gut taxa and weight are complex and may depend on host factors or intervention context.

Overall, the reviewed trials reinforce the hypothesis that interventions can rebalance the gut flora in children with obesity by favoring SCFA-producing and anti-inflammatory microorganisms and increasing microbial diversity. This aligns with broader evidence that enriching fiber and probiotic intake supports a healthier gut ecology [19].

Prebiotic and synbiotic supplementation represented the most frequently investigated microbiota-targeted strategies among the included interventions and were evaluated in most of the analyzed trials. Prebiotics (inulin, fructooligosaccharides, resistant starch, etc.) are non-digestible dietary fibers that are selectively fermented by the gut microbiota, leading to increases in beneficial bacterial groups including *Bifidobacterium* and *Lactobacillus*. This fermentation process also enhances the production of SCFAs, which are important metabolic end-products linked to host health [57]. These metabolic changes mediated weight effects: consuming 8–21 g/day of prebiotics for 2–6 months reduced BMI, waist circumference and inflammatory markers in pediatric obesity [57]. Moreover, an oligofructose preload lowered body fat percentage in obese children, with concomitant rises in stool *Bifidobacterium* DNA [59]. These findings echo prior reviews: Smolinska et al. concluded that targeting microbiota via prebiotics and probiotics significantly reduces intestinal permeability and improves gut barrier function in overweight individuals, partly via SCFA-driven pathways [58].

Synbiotics (combinations of probiotic strains and prebiotic substrates) were less studied but showed promising results in a subset of trials [60]. Nonetheless, not all formulations succeeded; a trial of lyophilized juçara pulp (a polyphenol-rich synbiotic) failed to alter microbiota or weight [61], underscoring that efficacy depends on the specific biotic.

Thus, the prebiotic/synbiotic trials reinforce the concept that selectively nourishing the gut flora can combat obesity. However, further research is necessary: dosage optimization and long-term outcomes are unclear. Future work should characterize which combinations (e.g., resistant starch + *Bifidobacterium*) are most synergistic, and for whom. The encouraging results to date support further exploration of precision biotics in pediatric obesity management.

Dietary changes (independent of added prebiotics) can also modulate the microbiome. Broadly, interventions emphasizing whole foods, high fiber, or reduced calories tended to shift the community toward a leaner profile. For example, high-fiber, Mediterranean-style diets led to an increased abundance of SCFA-producing genera like *Roseburia* and *Faecalibacterium*, while reducing Enterobacteriaceae [62]. Such diets are likely to function similarly to prebiotics, by providing fermentable substrates that favor health-associated microbes. Several studies reported that even without supplements, improved diet quality (e.g., more vegetables, legumes, and fermented foods) correlated with higher alpha diversity. Conversely, reducing simple sugars and fats tended to suppress pathobionts [63].

While few included studies tested strictly probiotic supplements, the broader nutrition literature indicates that fermented foods and dairy can introduce beneficial microbes (e.g., lactobacilli), though their survival and colonization are variable [64]. Overall, dietary interventions appear to nudge the pediatric gut microbiome in favorable directions, especially when focused on whole-plant foods and reduced ultra-processed intake. These findings are consistent with broader reviews arguing that positioning diet and lifestyle changes as microbiome modulators can aid obesity treatment [19].

These findings suggest that increased alpha diversity is not a universal marker of therapeutic benefit in pediatric obesity. In several dietary and combined lifestyle interventions, anthropometric or metabolic improvements occurred despite heterogeneous or absent changes in global diversity indices [38,39,40,41,42,43]. Moreover, baseline microbiome signatures were associated with metabolic response to butyrate supplementation, supporting the concept that the initial microbial configuration may influence intervention responsiveness [35]. Therefore, the effect of prebiotic or dietary interventions may depend on whether the child’s baseline microbiome contains the taxa and functional capacity required to metabolize the administered substrates and support short-chain fatty acid-producing networks. This may explain why some children show increases in diversity, whereas others demonstrate only taxon-specific, functional, or clinically relevant changes. These observations support the need for personalized nutrition strategies that consider baseline diet, microbiome composition, and metabolic phenotype before selecting microbiome-targeted interventions.

A methodological limitation of the available evidence is the frequent use of multicomponent interventions. In most included studies [36,38,39,40,41], dietary modification or nutritional counseling was delivered together with physical activity recommendations, supervised exercise programs, or broader lifestyle counseling. Therefore, the microbiome changes observed after these interventions cannot be attributed exclusively to diet or to a specific nutritional component. Exercise itself may influence gut microbiota composition and function through changes in energy expenditure, body composition, systemic inflammation, gut transit, and host metabolic status [44,45]. Consequently, the reported microbial shifts should be interpreted as the result of combined lifestyle interventions rather than as isolated diet-induced effects. This limits causal inference and makes it difficult to determine whether the gut microbiota changes were directly driven by dietary intake, physical activity, weight loss, or the interaction between these factors. Future studies should include clearly separated intervention arms, such as diet-only, exercise-only, combined diet–exercise, and control groups, to better define the independent and additive effects of each intervention component on the pediatric gut microbiome.

Also, the variability observed across studies may partly reflect fundamental differences between interventions based on isolated prebiotic compounds and those targeting overall dietary patterns. Supplementation with a single substrate, such as inulin or chitosan, is more likely to exert selective effects on specific microbial groups by providing defined fermentable substrates. In contrast, dietary modifications involving increased consumption of fiber-rich foods or restriction of free sugars influence the gut ecosystem through multiple mechanisms simultaneously. Whole foods provide not only diverse fermentable polysaccharides but also polyphenols, vitamins, minerals, and other bioactive constituents that can act synergistically to modulate microbial composition and function. This greater complexity may explain why comprehensive dietary interventions often produce broader, less predictable, and more heterogeneous microbiome responses than supplementation with isolated nutrients. Therefore, differences in the nature of the intervention should be considered when comparing the microbiological outcomes reported across pediatric obesity studies.

According to current data from the specialized literature, while the pediatric gut microbiota is responsive to dietary or lifestyle intervention, it is nevertheless not predictably programmable via such interventions alone [65]. Further research should focus on long-term, adequately sized pediatric RCTs with standardized microbiome and metabolism-related objectives and integrate multi-omic approaches to shed light on their therapeutic causality and relevance.

### Perspectives

Moving forward, large-scale, longitudinal studies are needed to establish causal links between microbiome modulation and obesity outcomes. Most existing trials were short-term; future research should track children over months or years to assess whether early microbial changes persist and translate into sustained weight improvement. Multi-center collaborations could recruit more diverse pediatric cohorts and standardize interventions and outcome measures. Adopting multi-omics approaches will be critical. Shotgun metagenomics should replace 16S to identify species- and strain-level changes, and to predict metabolic capacity. Metabolomic profiling of stool, blood, and urine would reveal shifts in SCFAs, bile acids, tryptophan metabolites, and other key molecules. Integrating transcriptomics or proteomics could pinpoint active microbial and host pathways during interventions.

Personalized strategies should also be explored. Given the heterogeneous responses observed, interventions may need tailoring to baseline microbiome or host phenotype. For example, a child lacking *Bifidobacterium* might benefit more from inulin than one who already has high levels. Integrating genomics (host genetics) and dietary habits could further refine personalization. Machine learning models could leverage large datasets to predict which children will respond to which microbiome therapies.

Clinically, the current findings imply that microbiome-targeted therapies could complement traditional obesity treatments in youth. Encouraging microbiota-friendly diets (high-fiber, plant-rich) and judicious use of probiotics/synbiotics may augment weight-management programs. However, it is premature to endorse specific supplements without more evidence. Physicians should continue emphasizing comprehensive lifestyle modifications (diet, activity) as first-line therapy, with microbiome modulation considered adjunctive. Practically, educating families about gut-healthy foods and limiting unnecessary antibiotics could also be beneficial based on mechanistic rationale.

In summary, interventions targeting the gut microbiome in pediatric obesity have shown biologically plausible benefits but remain an emerging field. To move toward clinical translation, we need rigorous, multidisciplinary research that links microbiome changes to tangible health gains. Long-term studies, advanced analytical techniques, and integration of behavioral science will be key. Ultimately, leveraging the gut microbiome may contribute to improving metabolic health in children; however, reaching this goal requires overcoming the methodological and conceptual challenges identified above.

**Figure 4:**
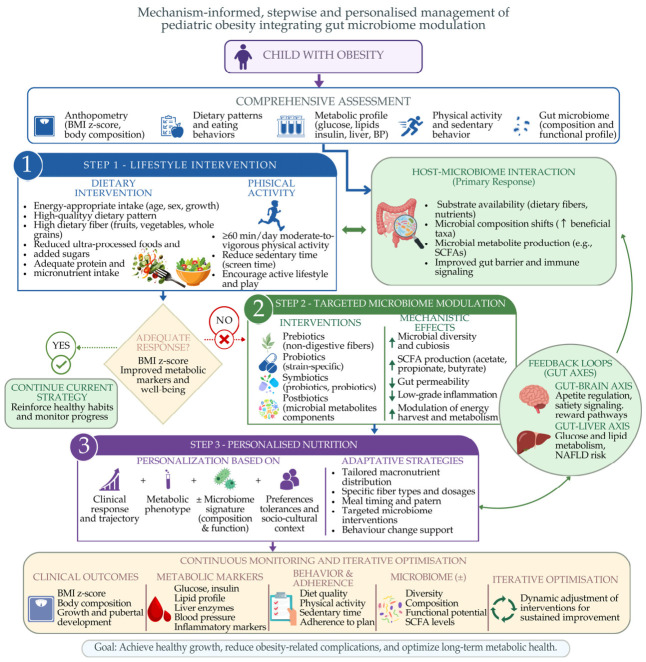
Mechanism-informed, stepwise and personalized management framework for pediatric obesity integrating gut microbiome modulation.

The framework (Figure 4) includes comprehensive assessment, lifestyle intervention, targeted microbiome modulation and personalized nutrition strategies, supported by continuous monitoring to improve metabolic health and long-term outcomes in children with obesity.

## 5. Conclusions

In conclusion, the present systematic review has shown that nutritional and lifestyle interventions in pediatric obesity are consistently linked to favorable anthropometric and metabolic outcomes. Dietary changes, in particular increased intake of fiber-rich, plant-based foods combined with caloric balance and physical activity, constitute the cornerstone of effective intervention.

Although many studies reported concurrent changes in gut diversity following such interventions, these findings were heterogeneous, context-dependent, and largely inconsistent across studies. Moreover, current evidence has not pointed to a causal relationship between microbiome alterations and clinical improvements. In fact, microbiota changes seem to represent secondary adaptations to dietary and behavioral changes. Methodological limitations include small sample sizes, short intervention length, study design heterogeneity, and methodological constraints, including the predominant use of 16S rRNA sequencing and a reliance on predictive functional analyses. These limitations may hinder the formulation of robust conclusions regarding the functional or clinical relevance of microbiome modulation.

Presently, microbiome-targeted strategies should be regarded as exploratory and adjunctive, rather than as primary therapy in pediatric obesity. Clinical management should therefore continue to prioritize evidence-based lifestyle interventions, while the role of the gut microbiome requires further clarification.

Future research should emphasize long-term, adequately powered randomized controlled trials, relying on standardized methodologies and multi-omics approaches, in order to assess the independent clinical significance of microbiome changes.

## Figures and Tables

**Table 1:** The main results of prebiotic intervention in pediatric obesity.

Author; Year; Country	Study Design	Population (Age, Diagnosis)	Type of Intervention	Duration of the Intervention	Main Results	Microbiome Analysis Method	**Comments**
Andriyas et al., 2025 [25]; Thailand	RCT (randomized controlled trial; double-blind, placebo-controlled trial)	*n* = 154 Children 7–15 years with obesity	Prebiotic inulin supplementation vs. Maltodextrin placebo vs. guidance on fiber intake; all received standard lifestyle counseling	6 months	↑ Gut–brain axis-related metabolites (putrescine, spermine, tyrosine); putrescine increased over time vs. placebo; metabolite changes correlated with gut microbiota composition, inflammatory markers, screen time, and satiety-related hormones	16S rRNA sequencing (Illumina) for microbiota; amino acids and biogenic amines were investigated using liquid chromatography–mass spectrometry; short-chain fatty acid (SCFA) by high-performance liquid chromatography (HPLC)	Focuses on microbiota-derived metabolites rather than clinical outcomes; findings largely associative, no direct causal link to weight loss demonstrated
Panichsillaphakit E et al., 2025 [26]; Thailand	RCT (double-blind, placebo-controlled)	*n* = 156, Children and adolescents 7–15 years with obesity	Prebiotic inulin supplementation (13 g/day from Thai Jerusalem artichoke) vs. isocaloric maltodextrin placebo vs. dietary fiber advice; all groups received standardized dietary and lifestyle counseling	6 months	Appetite modulation was noticed after inulin supplementation, significantly reduced emotional undereating compared with placebo; BMI z-score decreased in all groups without between-group differences	16S rRNA gene sequencing (Illumina)	Qualitative and quantitative changes in the gut microbiome pre- and post-intervention are not described
Aksornkitti et al., 2025 [27]; Thailand	RCT	*n* = 143, children 7–15 years with obesity	Inulin supplementation vs. isocaloric dextrin placebo vs. structured dietary fiber advice	6 months	↓ *Prevotella* and *Bacteroides*, ↑ Firmicutes; most participants switched between multiple enterotypes over time, indicating high intra-individual variability; no consistent intervention-specific effect attributable solely to inulin supplementation was demonstrated	16S rRNA gene amplicon sequencing (IlluminaMiSeq)	Study primarily addresses microbiome heterogeneity and temporal dynamics rather than clinical efficacy of inulin; microbiome outcomes are exploratory, with high variability during dietary interventions
Fatahi et al., 2025 [28]; Iran	RCT (double-blind, placebo-controlled clinical trial)	*n* = 61, adolescents 10–18 years with overweight or obesity	Chitosan supplementation (3 g/day) vs. maltodextrin placebo; both groups received standardized hypocaloric dietary advice and lifestyle counseling	12 weeks	Chitosan supplementation resulted in ↓ Firmicutes and Firmicutes/*Bacteroides* ratio, ↑ *Bacteroides* and *Akkermansia*, a significant reduction in BMI z-score compared with placebo; no significant increase in *Bifidobacterium* and borderline increase in *Lactobacillus*	Quantitative real-time PCR (qPCR) for selected bacterial taxa (Firmicutes, *Bacteroides*, *Akkermansia*, *Lactobacillus*, *Bifidobacterium*)	Microbiome assessment limited to selected taxa using qPCR; concurrent lifestyle intervention limits attribution of effects solely to chitosan; functional microbial outcomes not assessed
Visuthranukul C et al., 2024 [29]; Thailand	RCT (double-blind, placebo-controlled trial)	*n* = 143 Children 7–15 years with obesity	Prebiotic inulin supplementation (extracted from Thai Jerusalem artichoke) vs. Maltodextrin placebo vs. counseling on fiber intake; all received standard lifestyle counseling	6 months	↑ Alpha diversity, ↑ *Bifidobacterium*, *Blautia, Megasphaera* and butyrate-producing bacteria (*Agathobacter*, *Eubacterium coprostanoligenes*, *Subdoligranulum*); changes in functional pathways (proteasome, riboflavin metabolism); correlated with clinical and metabolic parameters only in inulin group	16S rRNA sequencing (Illumina) + functional inference via Phylogenetic Investigation of Communities by Reconstruction of Unobserved States 2 (PICRUSt2); fecal SCFA by HPLC	Clinical effects modest and mostly correlational

BMI = Body mass index, HPLC = High-performance liquid chromatography, PICRUSt = Phylogenetic Investigation of Communities by Reconstruction of Unobserved States, RCT = Randomized controlled trial, rRNA = ribosomal RNA, SCFA = Short-chain fatty acid, and qPCR = Quantitative polymerase chain reaction, ↑ indicates an increase and ↓ indicates a decrease in the abundance of the respective bacterial taxa.

**Table 2:** The main results of synbiotic intervention in pediatric obesity.

Author; Year; Country	Study Design	Population (Age, Diagnosis)	Type of Intervention	Duration of the Intervention	Main Results	Microbiome Analysis Method	Comments
Kilic Yildirim G et al., 2023 [30]; Turkey	RCT (double-blind, placebo-controlled clinical trial, single-center)	*n* = 54, children and adolescents 8–17 years with obesity	Synbiotic supplementation (*Lactobacillus acidophilus*, *Lacticaseibacillus rhamnosus*, *Bifidobacterium bifidum*, *Bifidobacterium longum*, *Enterococcus faecium*) + fructooligosaccharides (625 mg/day) vs. placebo; both groups received standardized hypocaloric diet and physical activity advice	12 weeks	↑ *Dialister* statistically significant for *Prevotella*, *Oscillospira* compared with baseline; ↑ *Prevotella*, *Coprococcus*, Lachnospiraceae (at genus level) and *Prevotella copri*, *Coprococcus eutactus*, *Ruminococcus* spp. (at species level) compared with baseline (mainly *Eubacterium dolichum*, *Lactobacillus ruminis*, *Clostridium ramosum*, *Bulleidia moorei*); synbiotic supplementation was associated with a greater reduction in BMI	16S rRNA gene sequencing (Illumina NovaSeq)	All participants received concurrent diet and exercise counseling, limiting isolation of synbiotic effects; microbiota analysis focused on bacteria only (no fungi, viruses or SCFA quantification); compliance with lifestyle intervention was self-reported; short intervention duration
Martínez-Martínez et al., 2022 [31]; Mexico	RCT (double-blind, three-arm interventional trial)	*n* = 12 probiotic group, *n* = 13 synbiotic-inulin group, *n* = 13 synbiotic-Agave fructans group; children 6–10 years with overweight or obesity	Fermented milk containing *Lactobacillus casei* Shirota administered in all groups. Comparison between: (1) probiotic alone, (2) probiotic + inulin (synbiotic), and (3) probiotic + *Agave salmiana* fructans (synbiotic)	6 weeks (intervention administered during school days only)	No significant differences in body weight or BMI were observed between groups. Synbiotic groups showed reductions in waist circumference and waist-to-height ratio. Microbiome analysis demonstrated significant differences in beta diversity between intervention groups. Genus-level changes included ↓ in *Blautia* and *Holdemanella* and ↑ in *Intestinibacter* and *Faecalibacterium*, with significant compositional shifts particularly in synbiotic formulations	16S rRNA gene amplicon sequencing (Ion PGM platform)	All intervention arms contained probiotic yogurt; therefore, the study evaluates synbiotic formulations rather than isolated prebiotic effects; small sample size and short intervention duration limit statistical power and generalizability; inclusion of small number of healthy children may confound obesity-specific microbiome effects

BMI = Body mass index, RCT = Randomized controlled trial, rRNA = ribosomal RNA, SCFA = Short-chain fatty acid, ↑ indicates an increase and ↓ indicates a decrease in the abundance of the respective bacterial taxa.

## Data Availability

The data presented in this study are available on request from the corresponding author. The data are not publicly available due to privacy and ethical reasons.

## Abbreviations

The following abbreviations are used in this manuscript:

A-CRD	Adjusted calorie-restricted diet
AIx	Augmentation index
ALT	Alanine aminotransferase
BC	Body composition
BMI	Body mass index
DEI	Diet and exercise intervention
PICRUSt	Phylogenetic Investigation of Communities by Reconstruction of Unobserved States
PRISMA	Preferred Reporting Items for Systematic Review and Meta-Analysis
RCT	Randomized controlled trial
rRNA	Ribosomal RNA
SCFA	Short-chain fatty acids
SNP	Single-nucleotide polymorphisms
SD	standard deviation
WHR	Waist-to-hip-ratio
qPCR	Quantitative real-time polymerase chain reaction
